# Two Distinct Mechanisms Govern RpoS-Mediated Repression of Tick-Phase Genes during Mammalian Host Adaptation by *Borrelia burgdorferi*, the Lyme Disease Spirochete

**DOI:** 10.1128/mBio.01204-17

**Published:** 2017-08-22

**Authors:** Arianna P. Grove, Dionysios Liveris, Radha Iyer, Mary Petzke, Joseph Rudman, Melissa J. Caimano, Justin D. Radolf, Ira Schwartz

**Affiliations:** a Department of Microbiology and Immunology, New York Medical College, Valhalla, New York, USA; b Department of Medicine, UConn Health, Farmington, Connecticut, USA; c Department of Pediatrics, UConn Health, Farmington, Connecticut, USA; d Department of Molecular Biology and Biophysics, UConn Health, Farmington, Connecticut, USA; e Department of Genetics and Genomic Science, UConn Health, Farmington, Connecticut, USA; f Department of Immunology, UConn Health, Farmington, Connecticut, USA; NIAID, NIH

**Keywords:** *Borrelia burgdorferi*, Lyme disease, RpoS, sigma factors, transcriptional repression

## Abstract

The alternative sigma factor RpoS plays a key role modulating gene expression in *Borrelia burgdorferi*, the Lyme disease spirochete, by transcribing mammalian host-phase genes and repressing σ^70^-dependent genes required within the arthropod vector. To identify *cis* regulatory elements involved in RpoS-dependent repression, we analyzed green fluorescent protein (GFP) transcriptional reporters containing portions of the upstream regions of the prototypical tick-phase genes *ospAB*, the *glp* operon, and *bba74*. As RpoS-mediated repression occurs only following mammalian host adaptation, strains containing the reporters were grown in dialysis membrane chambers (DMCs) implanted into the peritoneal cavities of rats. Wild-type spirochetes harboring *ospAB*- and *glp-gfp* constructs containing only the minimal (−35/−10) σ^70^ promoter elements had significantly lower expression in DMCs relative to growth *in vitro* at 37°C; no reduction in expression occurred in a DMC-cultivated RpoS mutant harboring these constructs. In contrast, RpoS-mediated repression of *bba74* required a stretch of DNA located between −165 and −82 relative to its transcriptional start site. Electrophoretic mobility shift assays employing extracts of DMC-cultivated *B. burgdorferi* produced a gel shift, whereas extracts from RpoS mutant spirochetes did not. Collectively, these data demonstrate that RpoS-mediated repression of tick-phase borrelial genes occurs by at least two distinct mechanisms. One (e.g., *ospAB* and the *glp* operon) involves primarily sequence elements near the core promoter, while the other (e.g., *bba74*) involves an RpoS-induced transacting repressor. Our results provide a genetic framework for further dissection of the essential “gatekeeper” role of RpoS throughout the *B. burgdorferi* enzootic cycle.

## IMPORTANCE

*Borrelia burgdorferi*, the Lyme disease spirochete, modulates gene expression to adapt to the distinctive environments of its mammalian host and arthropod vector during its enzootic cycle. The alternative sigma factor RpoS has been referred to as a “gatekeeper” due to its central role in regulating the reciprocal expression of mammalian host- and tick-phase genes. While RpoS-dependent transcription has been studied extensively, little is known regarding the mechanism(s) of RpoS-mediated repression. We employed a combination of green fluorescent protein transcriptional reporters along with an *in vivo* model to define *cis* regulatory sequences responsible for RpoS-mediated repression of prototypical tick-phase genes. Repression of *ospAB* and the *glp* operon requires only sequences near their core promoters, whereas modulation of *bba74* expression involves a putative RpoS-dependent repressor that binds upstream of the core promoter. Thus, Lyme disease spirochetes employ at least two different RpoS-dependent mechanisms to repress tick-phase genes within the mammal.

## INTRODUCTION

In most bacteria, modulation of gene expression occurs via selective promoter recognition and productive transcription initiation. All bacteria encode a housekeeping sigma factor (e.g., sigma 70 [σ^70^] in Gram-negative bacteria) that is responsible for recognition of the vast majority of promoters by RNA polymerase (RNAP) holoenzyme (1, 2). In addition, nearly all bacteria have alternative sigma factors that regulate the expression of a subset of genes in response to specific environmental, physiological, and/or metabolic cues. Most evidence suggests that housekeeping and alternative sigma factors interact with RNAP holoenzyme in a similar manner (1, 2). Under homeostatic growth conditions, σ^70^ is substantially more abundant than alternative sigma factors and, consequently, directs the vast majority of transcription. However, during growth transitions (e.g., entry into stationary phase) and/or exposure to specific, often stressful, environmental stimuli, the levels and activities of alternative sigma factors increase, enabling them to compete with σ^70^ for apo-RNAP and thereby direct transcription of genes whose products promote adaptation to the altered physiological state or environmental milieu (2).

*Borrelia burgdorferi*, the etiologic agent of Lyme disease, is maintained in nature within an enzootic cycle involving small reservoir hosts, such as rodents and an ixodid tick vector (3–6). As there is no transovarial transmission of *B. burgdorferi*, larvae must acquire the spirochete by feeding on an infected host (7, 8). *B. burgdorferi* is retained in the tick midgut during the molt into the nymphal stage. During the nymphal blood meal, there is a replicative burst of *B. burgdorferi* within the midgut, and spirochetes transition from a nonmotile to motile state, enter the hemocoel, migrate to the salivary glands, and are transmitted to the next host (9–12). These drastic changes in environmental conditions require the spirochete not only to adjust the expression of colonization factors and other surface molecules but also to alter its metabolic state in response to the changing nutrient profile (13, 14).

The *B. burgdorferi* genome encodes only three sigma factors, a housekeeping σ^70^ and the alternative sigma factors RpoN and RpoS (6, 15–17). The consensus σ^70^ promoter and behavior of RNAP in *B. burgdorferi* are thought to mirror their well-studied counterparts in *Escherichia coli* (18, 19). Indeed, a recent global analysis of *B. burgdorferi* promoters demonstrated that the consensus −10 region (TATAAT), the minimal core promoter element, is essentially the same as in *E. coli*; however, no strong consensus was observed for the −35 region in *B. burgdorferi* (20). Examination of the upstream regions for RpoS-induced genes suggested that *B. burgdorferi* RpoS recognizes an extended −10 sequence that is distinct from the σ^70^ consensus promoter (21, 22). Seminal studies by Norgard and coworkers demonstrated a link between the Hk2-Rrp2 two-component system (TCS), RpoN, and RpoS (23, 24). At the onset of the nymphal blood meal, Rrp2 and RpoN, along with BosR, activate the expression of *rpoS*, which in turn upregulates the expression of genes required for tick-to-mammal transmission (*cdr* and *mlp4* and -*5*) and/or virulence within the mammal (*ospC*, *dbpBA*, *bbk32*, and *bba34*) (16, 17, 22, 23, 25). Global transcriptome analyses of wild-type and *rpoS* mutant strains under mammalian host-like conditions defined 104 genes that are induced by RpoS *in vivo*, many encoding proteins of unknown function (22).

Following transmission to the mammal, RpoS is also essential for repression of σ^70^-transcribed tick-phase genes (12, 22, 26–28). On this basis, RpoS has been referred to as a “gatekeeper” for the reciprocal expression of genes required for either mammalian infection or maintenance in ticks (22). While numerous studies have investigated the nature of RpoS-dependent transcription in *B. burgdorferi* (6, 16, 25, 29–35), virtually nothing is known regarding the mechanism(s) underlying repression by RpoS. This is, to a large extent, due to the fact that RpoS-mediated repression does not occur *in vitro* under experimental conditions (i.e., following temperature shift) in which RpoS-dependent genes are known to be induced. Instead, repression by RpoS requires environmental stimuli that are unique to the mammalian host milieu (22, 26). In the past, we have circumvented this limitation using the dialysis membrane chamber (DMC) peritoneal implant model to generate mammalian host-adapted *B. burgdorferi* (22, 28, 36, 37). Among the cohort of tick-phase genes subject to RpoS-mediated repression are the *glp* operon (*bb0240* to -*243*), *ospAB* (*bba15/16*), and *bba74*. To better understand the molecular mechanism(s) underlying RpoS-dependent repression, we used a series of green fluorescent protein (GFP) transcriptional reporter constructs, in conjunction with our DMC cultivation system, to explore the promoter elements of these three prototypical RpoS-repressed tick-phase loci. Our results suggest that *B. burgdorferi* employs at least two different mechanisms for RpoS-mediated repression within the mammal.

## RESULTS

### Comparison of *ospAB*, *bba74*, and *glp* operon promoter regions reveals few common regulatory motifs.

**FIG 1:**
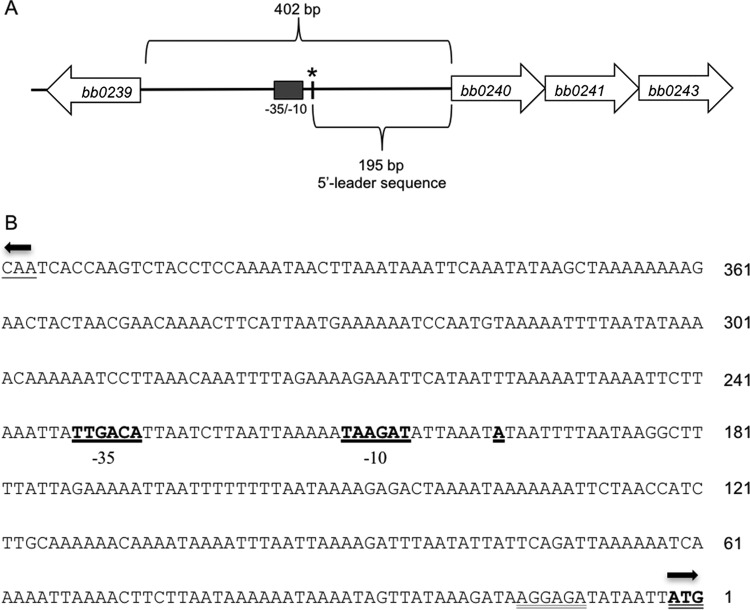
Identification of the *glp* operon transcriptional start site and promoter by 5′ RACE. (A) Schematic diagram of the *glp* operon region. The asterisk denotes the transcriptional start site (+1). (B) Sequence of *glp* operon TSS and promoter. The TSS and −35/−10 promoter elements are in boldface and underlined; the *glpF* (*bb0240*) translational start site is in boldface and denoted by a double underline; the putative Shine-Dalgarno site is indicated by double underline; the *bb0239* translational start site is underlined. Arrows indicate direction of translation.

We began by comparing the sequences upstream of *ospAB*, *glpF*, and *bba74*, three prototypical tick-phase genes, whose expression is known to be downregulated by RpoS *in vivo*. The transcriptional start sites (TSSs) for *ospA* and *bba74* during growth *in vitro* have been previously reported (27, 38). 5′ rapid amplification of cDNA ends (RACE) was performed to definitively map the *glpF* TSS. Surprisingly, the TSS was located 195 bp upstream from the translation initiation site (Fig. 1). Consistent with these results, Adams et al. (20) recently reported the existence of a 195-bp untranslated leader sequence (UTR) in the *glp* operon by global 5′-end mapping and Northern blotting.

**FIG 2:**
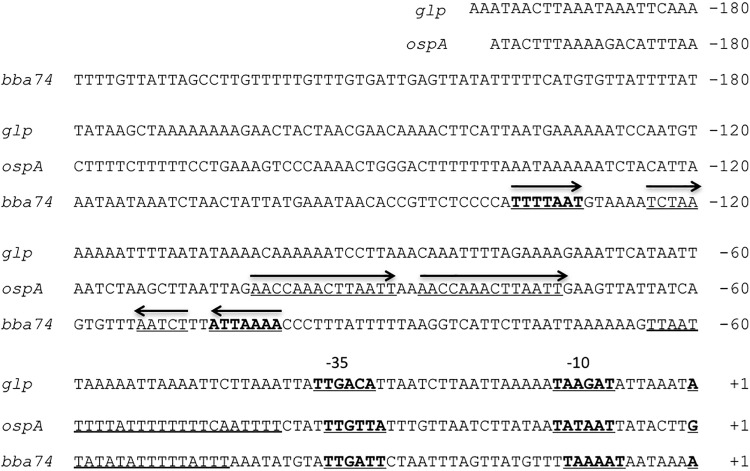
Alignment of *glp* operon, *ospAB*, and *bba74* upstream sequences. Sequences are numbered relative to the TSS (designated +1). −35/−10 promoter elements are in bold and underlined. T-rich regions in *ospAB* and *bba74* are indicated by double underline. A direct repeat in *ospA* (−102 to −73) is indicated by forward arrows; the inverted repeat in *bba74* (−137/−131 to −106/−100) is in bold and underlined and indicated by inverted arrows; the reverse repeat in *bba74* (−124/−120 to −113/−109) is underlined and indicated by inverted arrows.

Sequence alignment revealed that the extended promoter regions for *ospAB*, the *glp* operon, and *bba74* differ at the primary sequence and secondary structure levels (Fig. 2). Multiple groups have examined the *cis* regulatory elements in the upstream region of *ospA* (26, 39, 40). Collectively, they suggested that a T-rich region is required for maximum expression *in vitro*. The region upstream of the *bba74* TSS also contains a T-rich region, but no such element is discernible for the *glp* operon (Fig. 2). In addition, the *ospAB* upstream region contains a direct repeat and the *bba74* extended promoter contains an inverted repeat. No obvious secondary structure motifs are discernible in the *glp* promoter upstream region (Fig. 2). The lack of any obvious shared upstream primary sequences or predicted secondary structure motifs raised the possibility that multiple mechanisms of RpoS-mediated repression are operative in *B. burgdorferi*.

### Repression of *ospAB* and *glp* operon expression occurs by a mechanism different from that regulating *bba74.*

**FIG 3:**
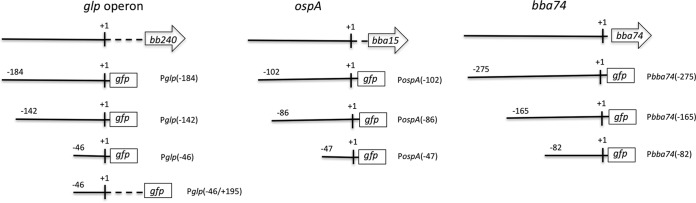
Schematic representation of promoter-*gfp* fusions. Promoter-*gfp* transcriptional reporters with decreasing lengths of the upstream sequence were generated. The shortest fusions contain the core promoters (−10/−35 plus minimal additional sequence). “+1” denotes the TSS. The region upstream of the TSS is indicated by a solid line, and the 5′ untranslated region in the *glp* operon is depicted by a dashed line (drawn approximately to scale). Except for P*glp*(−46/+195), all reporter constructs exclude the 5′ untranslated leader regions. Designated names for the constructs are to the right of each construct.

**FIG 4:**
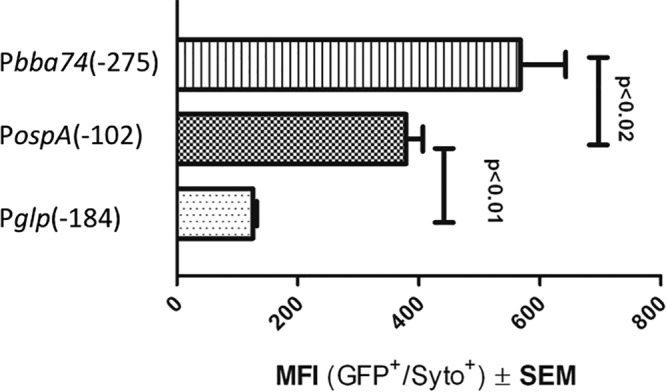
Transcriptional activities for full-length promoters vary during *in vitro* growth at 37°C. *B. burgdorferi* B31 5A18 NP1 transformed with the indicated promoter-*gfp* fusion constructs was grown in BSK medium at 37°C. Mean fluorescence intensity (MFI) of GFP expression by each construct was measured by flow cytometry. Data for each reporter represent a minimum of three independent experiments.

In order to identify potential *cis* regulatory elements, GFP transcriptional reporter constructs containing various amounts of sequence upstream of the TSSs of *ospAB*, *bba74*, and the *glp* operon were transformed into B31 5A18 NP1 (Fig. 3; see also Fig. 9). For each gene, we first examined GFP expression by the construct containing the greatest amount of upstream sequence during *in vitro* growth at 37°C. P*bba74*(−275) elicited the highest average mean fluorescence intensity (MFI) [1.5-fold and 4.5-fold greater than the P*ospA*(−102) and P*glp*(−184), respectively]. The MFIs for both P*ospA*(−102) and P*bba74*(−275) were significantly greater than that of P*glp*(−184) (Fig. 4).

**FIG 5:**
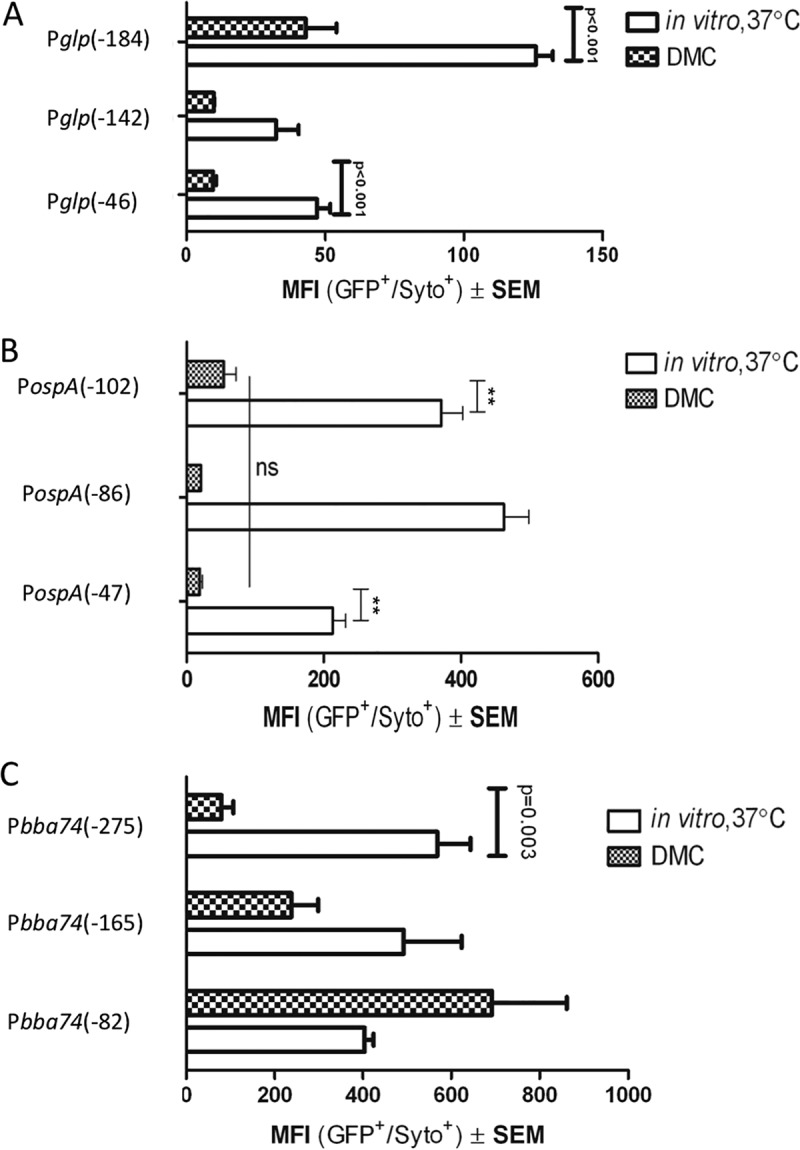
Expression from promoter*-gfp* reporters during *in vitro* and DMC cultivation. The promoter*-gfp* reporters were cultivated either at 37°C (open bars) or in DMCs (checkered bars). GFP MFIs were measured by flow cytometry. (A) *glp* operon; (B) *ospAB*; (C) *bba74*. **, *P* < 0.01; ns, not significant. Data for each reporter construct represent a minimum of two independent biological replicates, except for P*ospA*(−86), for which only one biological sample was recovered from DMC cultivation.

We next compared GFP expression by full-length and 5′ truncations for each upstream region following temperature shift *in vitro* and cultivation within DMCs. As shown in Fig. 5A, all of the P*glp* reporters produced measurable expression of GFP during *in vitro* growth, with P*glp*(−184) eliciting the highest MFI. When 42 bp was removed [Pglp(−142)], expression of GFP *in vitro* decreased significantly (*P* = 1.31 × 10^−6^), suggesting that this region may contain an activator/enhancer site. Deletion of an additional 96 bp [Pglp(−46)] had no significant effect. Although the P*glp*(−184) reporter drove measurable GFP expression within DMCs, it was markedly lower (*P* < 0.001) than that during *in vitro* growth (Fig. 5A), consistent with previous studies for the native *glp* operon (12, 22). Interestingly, deletion of the same 42 bp that resulted in decreased expression of GFP *in vitro* virtually eliminated expression *in vivo* [compare P*glp*(−184) to P*glp*(−142)]. Thus, complete repression of the *glp* operon *in vivo* is mediated via sequences in the vicinity of the core promoter (i.e., −10/−35).

As shown in Fig. 5B, the full-length and truncated P*ospA*-*gfp* reporters expressed measurable levels of GFP *in vitro* at 37°C. We observed a significant decrease in GFP expression for P*ospA*(−47) relative to P*ospA*(−102) and P*ospA*(−86), suggesting that the region between −86 and −47 may contain a positive regulatory element (Fig. 5B). Deletion of these 39 bp removes the poly(T) tract (Fig. 2) that Sohaskey et al. (39) suggested was responsible for enhanced expression of *ospA in vitro*. When *B. burgdorferi* strains harboring these P*ospA* reporters were grown in DMCs, all three exhibited a significant decrease in GFP expression (Fig. 5B). Importantly, there were no significant differences in GFP expression between the P*ospA* reporters, suggesting that, as with the *glp* promoter, sequences near the core −35/−10 sequence motif are sufficient for repression of *ospAB*.

GFP expression driven by P*bba74*(−275) was also significantly repressed within DMCs (Fig. 5C). In contrast to the P*glp* and P*ospA* reporters, removal of a region between −165 and −82 (relative to the *bba74* TSS [Fig. 2]) abrogated repression of the P*bba74* reporter in DMCs, whereas it had no discernible effect on expression *in vitro* (Fig. 5C). This region, therefore, may contain an effector site required for repression *in vivo*.

### Repression of *ospAB* and the *glp* operon during mammalian host adaptation is RpoS dependent.

**FIG 6:**
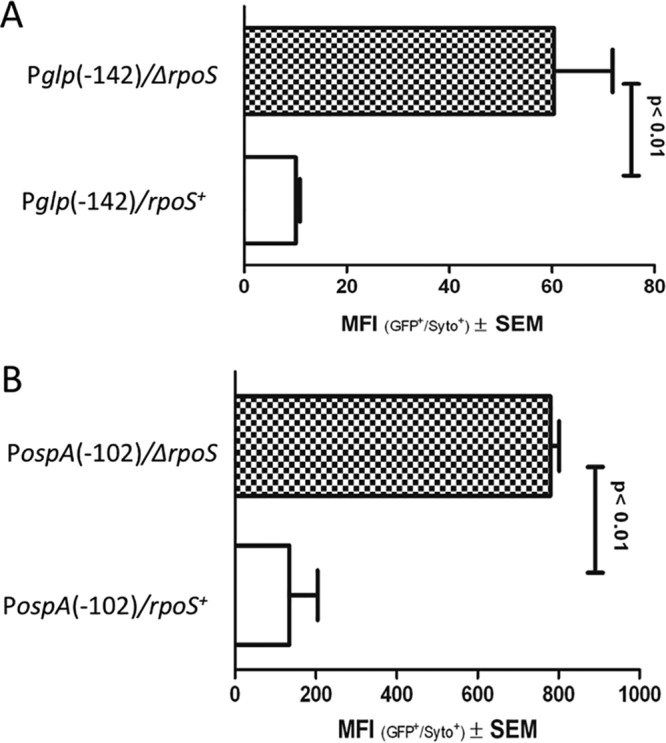
Transcriptional repression in DMCs is dependent on RpoS. P*glp*(−142)-*gfp* (A) and P*ospA*(−102)-*gfp* (B) were transformed into either wild-type B31 5A18 or the Δ*rpoS* mutant (in a strain 297 background) and cultivated in DMCs. GFP MFI was measured by flow cytometry.

In order to determine if repression of the P*glp* and P*ospA* reporters during mammalian host adaptation is RpoS dependent, we transformed the P*glp*(−142) and P*ospA*(−102) constructs into a *B. burgdorferi* strain 297 Δ*rpoS* mutant (25) and measured GFP in the transformants following cultivation in DMCs. Note that the RpoS mutant employed in these studies was on a strain 297 background, whereas the wild type was a B31 strain (B31 5A18). This was necessitated by the unavailability of a strain B31 Δ*rpoS* mutant at the time that these experiments were performed. Several factors suggest that this should not represent a problem. First, RpoS regulon expression levels have been shown to be similar between the B31 and 297 strains (22, 28). In addition, we sequenced the regions upstream of the transcription start sites for *glpF* and *bba74* in strain 297. For *glpF*, there is an A→T change at position −3, and for *bba74*, there is a G→A change at position −253 and an A→G change at position −53. Comparison of the B31 and 297 sequences deposited in GenBank for the region upstream of *ospA* revealed a single G→A change at position −26. None of these single nucleotide polymorphisms (SNPs) are likely to affect either expression or RpoS-mediated regulation. In accord with previous findings for the native genes (12, 22, 27), we saw substantially higher expression of GFP (i.e., derepression) for both reporters in the absence of RpoS (Fig. 6). Moreover, these results indicate that the −46/−47 to −1 regions of both genes are sufficient for RpoS-dependent repression.

### Repression of *bba74* expression during growth in DMCs requires an RpoS-dependent factor.

**FIG 7:**
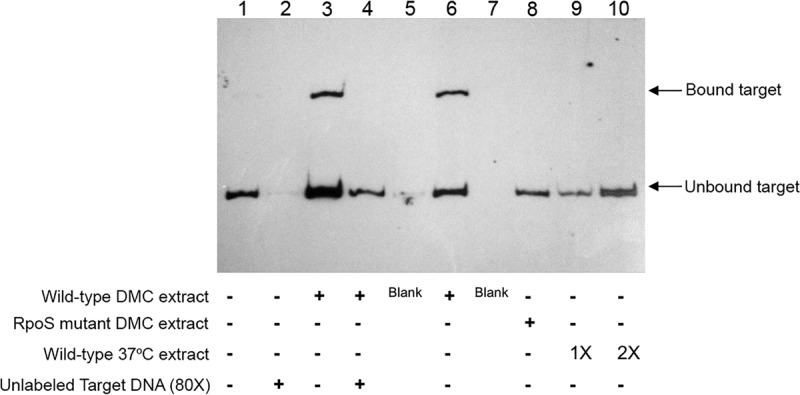
Electrophoretic mobility shift assays demonstrating RpoS dependence for repression of *bba74* expression during cultivation in DMCs. Binding reactions were performed in a total volume of 20 μl containing 50 ng of poly(dI⋅dC), 50 fmol biotin-labeled target DNA, and 3 μg of protein lysate (where indicated). Lane 1, no additions; lane 2, 4 pmol of unlabeled target DNA; lanes 3 and 6, wild-type extract from DMC-cultivated spirochetes; lane 4, wild-type extract from DMC-cultivated spirochetes plus 4 pmol unlabeled target DNA; lane 8, extract from RpoS mutant cultivated in DMCs; lane 9, 3 μg wild-type extract from spirochetes cultivated *in vitro* at 37°C; lane 10, 6 μg wild-type extract from spirochetes cultivated *in vitro* at 37°C.

Previously, we reported that repression of the native *bba74* gene within DMCs is RpoS dependent (22, 27). Above, we showed that repression of *bba74 in vivo* requires upstream sequences located between −165 and −82 (Fig. 5C). These results led to the prediction that this region contains the binding site for an RpoS-dependent repressor. Despite multiple attempts, we were unable to obtain Δ*rpoS* transformants harboring P*bba74*-*gfp* constructs to test this conjecture. As an alternative approach, we employed electrophoretic mobility shift assays (EMSAs) with a DNA fragment encompassing 275 bp upstream of the *bba74* TSS (Fig. 7). Incubation of this fragment with a cell extract prepared from wild-type spirochetes cultivated in DMCs resulted in a mobility shift (Fig. 7, lanes 3 and 6), which was completely inhibited by the addition of an 80-fold excess of unlabeled target DNA (lane 4). Importantly, incubation of the target DNA with a cell extract prepared from DMC-cultivated Δ*rpoS* mutant did not produce a shifted product (lane 8). Incubation with a cell extract from wild-type *B. burgdorferi* grown *in vitro* at 37°C also failed to produce a shift (lanes 9 and 10). These findings not only confirm, as expected, that repression of *bba74* expression is RpoS dependent (27), but they also indicate that repression appears to be mediated by a factor that is produced or functions only in response to mammalian host-specific signals.

### Role of 5′ UTR in regulation of *glp* operon expression.

**FIG 8:**
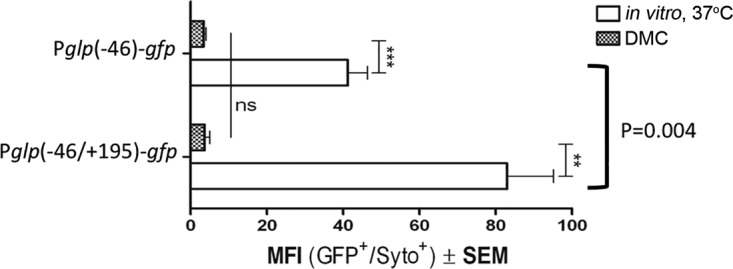
The 5′ UTR does not have a role in the transcriptional repression of the *glp* operon in DMCs. P*glp*(−46) (containing only core promoter) and P*glp*(−46/+195) (containing minimal promoter and 5′ UTR) were cultivated *in vitro* at 37°C or in DMCs. GFP MFI was measured by flow cytometry. **, *P* < 0.01; ***, *P* < 0.001; ns, not significant.

5′ UTRs have been shown to control gene expression transcriptionally and posttranscriptionally (41). To elucidate the possible contribution of the leader sequence to *glp* expression, we constructed a GFP reporter containing the core promoter plus the 5′ UTR, P*glp*(−46/+195). The presence of the leader sequence resulted in significantly higher GFP expression *in vitro* compared with the minimal promoter (*P* = 0.004) (Fig. 8). However, the two reporters expressed GFP at similarly low levels within DMCs (*P* = 0.821) (Fig. 8). Thus, the 5′ UTR does not appear to play a role in the repression of the *glp* operon *in vivo*. The enhanced expression in the presence of the 5′ UTR *in vitro* may be the result of either stabilization of the longer transcript or enhancement of translation efficiency, but the precise mechanism remains to be elucidated.

## DISCUSSION

The cytosolic concentrations of σ factors typically exceed those of RNAP (1, 42). On this basis, repression of σ^70^-dependent gene expression by alternative σ factors has generally been thought to result from competition for limiting apo-RNAP (42–45). Alternatively, RpoS may control the expression of one or more repressor proteins or regulatory RNAs that is/are not induced or not fully active until spirochetes are within the mammal (22, 26, 46). A third possibility is competition between σ^70^-RNAP and RpoS-RNAP holoenzymes for promoter binding. In a recent study, Levi-Meyrueis et al. (47) demonstrated that a *Salmonella enterica* serovar Typhimurium RpoS mutant defective in DNA binding (but capable of forming RNAP holoenzyme) had a global expression profile highly similar to that of an RpoS-deficient strain. Further, RpoS-dependent repression of selected genes was not solely the result of σ factor competition for binding to apo-RNAP. The authors concluded that RpoS-mediated repression resulted from occlusion of certain σ^70^ promoters by RpoS-RNAP holoenzyme, thereby reducing σ^70^-dependent transcription initiation (47). Our results suggest that RpoS-dependent repression of tick-phase genes in *B. burgdorferi* can occur by at least two different mechanisms. Repression of *ospAB* and *glp* operon transcription most likely occurs due to increased competition between σ^70^-RNAP and RpoS-RNAP holoenzymes for binding to the respective σ^70^ promoters. In contrast, repression of *bba74* appears to result from RpoS-induced synthesis or activation of a repressor protein that binds upstream of the core promoter, blocking σ^70^-RNAP holoenzyme access to the promoter.

Although our promoter fusion experiments were designed to shed light specifically on the mechanism of RpoS-mediated repression, additional potential regulatory insights were revealed during the study. *ospA* activation has been examined by multiple groups (26, 39, 40). Consistent with the requirement for the T-rich region for full *ospA* expression *in vitro* (39, 40), the *ospA* −86 GFP construct [P*ospA*(−86)] containing the T-rich region had significantly higher expression *in vitro* than the *ospAB* core promoter construct [P*ospA*(−47)] (Fig. 5B). Xu et al. reported that a large direct repeat is required for maximum expression *in vitro* (40). The −102 *ospA* reporter [P*ospA*(−102)] contains the intact repeat, and this region is disrupted in the −86 *ospA* reporter construct [P*ospA*(−86)] (Fig. 2). GFP expression did not significantly change in the shorter construct (Fig. 5B), suggesting that the direct repeat is not required for maximum *in vitro* expression. Importantly, GFP expression was not significantly different among the *ospA* reporters during growth in DMCs (Fig. 5B). Thus, the upstream *cis* elements required for full *ospAB* expression during cultivation *in vitro* cannot overcome the repression that occurs during mammalian host adaptation.

Recently, Li, Liang, and colleagues (48, 49) identified two putative binding sites for BosR near the *ospA* promoter and proposed that BosR functions as an *ospA* repressor. One of the BosR binding sites is located downstream of the TSS and, therefore, is not included in our P*ospA* reporters, whereas the second site overlaps the P*ospA* −10 region. Thus, BosR may contribute to the reduced expression of our P*ospA* reporters in DMCs (Fig. 5B). If, however, BosR is able to bind directly to the *ospA* promoter, why is RpoS also required for repression *in vivo*? One possible explanation is that BosR and RpoS act cooperatively to block transcription by σ^70^-RNAP holoenzyme at the *ospA* core promoter. While analysis of our *ospA* reporters in BosR- and RpoS-deficient backgrounds could be informative, these studies are complicated by the fact that Δ*bosR* mutants also lack RpoS (31, 50). The only way to definitively establish whether BosR functions as a direct repressor of *ospAB* is to conduct studies of *B. burgdorferi* constructs in which expression of *rpoS* is independent of BosR under mammalian host conditions.

Glycerol utilization has been shown to be a fitness requirement for *B. burgdorferi* during the tick phase (12, 51) and is apparently dispensable during mammalian infection (12, 28). The *glp* operon is reciprocally regulated by the RpoN-RpoS and Hk1-Rrp1 TCS (22, 46, 51, 52). Rrp1 is a diguanylate cyclase that catalyzes production of cyclic di-GMP, whose effect is mediated through PlzA, the only cyclic di-GMP binding protein in *B. burgdorferi* (46, 53). *glp* operon expression is severely reduced in Rrp1 and PlzA mutants (46, 51, 52). Recently, Rel_Bbu_ has also been shown to be an activator of *glp* operon expression (54, 55).

Comparison of GFP expression from P*glp*(−184) and P*glp*(−142) during *in vitro* cultivation at 37°C reveals that the 42-bp region between −184 and −142 is required for maximal expression. Further, removal of this 42-bp sequence also results in decreased expression during growth in DMCs (Fig. 5A). This suggests that the −184 to −142 region may represent an enhancer site. *glp* operon expression in the mammal appears to be a composite of two opposing regulatory mechanisms—induction that is partially dependent on an enhancer site (−184 to −142) and repression mediated by RpoS at the core promoter (−46 to −1) (Fig. 5A). It is unclear whether Hk1/Rrp1 (c-di-GMP) and/or Rel_Bbu_ is involved in mediating interaction with this putative enhancer sequence; however, further elucidation of the mechanism for *glp* operon induction could potentially be assessed by measuring GFP expression driven by P*glp*(−184) and P*glp*(−142) in Rrp1, PlzA, or Rel_Bbu_ mutant backgrounds.

In contrast to *ospAB* and the *glp* operon, a sequence between nucleotides −165 and −82 upstream of the *bba74* TSS is required for RpoS-dependent repression; removal of this region resulted in derepression under mammalian host-adapted conditions (Fig. 5C). An EMSA using the 283 bp upstream of the *bba74* TSS as a target with cell extracts isolated from *B. burgdorferi* propagated in DMCs suggests the presence of a protein that specifically binds to this DNA region. This putative DNA-binding protein is present or active only in extracts from host-adapted spirochetes, consistent with the notion that its synthesis is dependent on RpoS (Fig. 7). The precise binding site at which this putative repressor binds is under investigation; however, a careful scan of the *bba74* upstream region reveals several unusual sequence features that may play a role in the putative repressor protein binding (Fig. 2). In particular, there is an inverse repeat at nucleotides −138 to −132 (5′..TTTTAAT..3′) and −107 to −101 (5′..ATTAAAA..3′), both of which are flanked by CCC sequences that may be used to stabilize a potential stem-loop structure formed by these repeats. In addition, positions −125 to −121 (5′..TCTAA..3′) and −114 to −110 (5′..AATCT..3′) are reverse repeats that ensure that no alternative stem can be formed in this region of the promoter. Although transcriptional repressors typically bind closer to their cognate promoters, global bioinformatic analyses of transcription factors in *E. coli* and *Bacillus subtilis* identified numerous instances of repressor binding at a greater distance upstream (56, 57). The putative RpoS-dependent *bba74* repressor is likely encoded by a gene induced by RpoS during mammalian growth.

## MATERIALS AND METHODS

### Identification of *glp* operon transcriptional start site.

*B. burgdorferi* B31 A3 (58) was cultivated initially in Barbour-Stoenner-Kelly medium II (BSK-II) at 25°C to a density of 1 × 10^7^ cells/ml. Cultures were diluted to 3,000 cells per ml into 10 ml of fresh BSK-II and grown to late logarithmic phase (~1 × 10^8^ cells/ml) at 37°C. Cells were centrifuged for 15 to 20 min at 8,000 × *g*, washed three times in 1× phosphate-buffered saline (PBS), and resuspended in 10 μl of 1× PBS. RNA was extracted using the ToTALLY RNA kit (Ambion, Foster City, CA) according to the manufacturer’s protocol. The RNA pellet was resuspended in 30 μl of nuclease-free water and treated twice with DNase using the Ambion DNA-free kit (Ambion). Isolated RNA was stored at −80°C in the presence of RNasin until further use.

The transcriptional start site for *glpF*, the first gene in the *glp* operon, was identified using the 5′ RACE System for Rapid Amplification of cDNA Ends kit, version 2.0 (Invitrogen, Carlsbad, CA). First-strand cDNA synthesis was accomplished using the gene-specific primer BB0240_race2 (Table 1). A 2.5-pmol amount of BB0240_race2 primer was added to 5 μg of RNA and diethyl pyrocarbonate (DEPC)-treated water in a final volume of 15.5 μl, and synthesis of first-strand cDNA, terminal deoxynucleotidyl transferase (TdT) tailing, and second-strand synthesis by PCR using BB0240_race/Abridged Anchor Primer pair (Table 1) were performed according to the manufacturer’s instructions. cDNA recovery was checked by PCR amplification using the BB0243F3/BB0240_race primer pair (Table 1), and PCR products were analyzed on a 1% agarose gel to confirm amplification of the 5′ RACE products. The purified amplicons were cloned into pGEM-T (Promega, Madison, WI) and transformed into *E. coli* DH5α followed by blue/white selection on LB agar plates containing 100 μg/ml ampicillin. Selected clones were confirmed by PCR using BB0240_race/Abridged Anchor Primer. Inserts from confirmed clones were amplified by PCR using the pGEM-T universal forward and reverse primers and sequenced (Genewiz, South Plainfield, NJ).

**TABLE 1:** Oligonucleotides utilized in this study

Primer name	Sequence (5′–3′)	Purpose
BB0240_race2	TGCTAACAGCTGGGTTTAGGTGTG	PCR; 5′ RACE
BB0240_race	TCCTGGTATTTCGGGACTTGAGGA	PCR; 5′ RACE
BB0240F3	CAGATTAAAAAATCAAAAATTA	PCR; 5′ RACE
AC240ΔF1	CAAATATAAGCTAAAAAAAAGAAC	PCR; GFP fusion
AC240ΔF2	CATTAATGAAAAAATCCAATG	PCR; GFP fusion
AC240ΔF3	CTTAAATTATTGACATTAATC	PCR; GFP fusion
240TSSrev	TATTTAATATCTTATTTTTAATTAAG	PCR; GFP fusion
BB0240fullR	CTTTATAACTATTTTATTTTTTATTAAG	PCR; GFP fusion
OspAΔF2	GAACCAAACTTAATTAAAACC	PCR; GFP fusion
OspAΔF3	AACCAAACTTAATTGAAG	PCR; GFP fusion
OspAΔF4	CAATTTTCTATTTGTTATTTG	PCR; GFP fusion
OspAfullR	CTTAATACAAGTATAATTATATTATAAG	PCR; GFP fusion
A74pfullF	GGCAATGTTTGCTAAGGTG	PCR; GFP fusion; EMSA
A74pfullR	CATTTATTTTTATTATTTTAAAAC	PCR; GFP fusion
A74ΔF2	CTATTATGAAATAACACCG	PCR; GFP fusion
A74ΔF3	CATTCTTAATTAAAAAAG	PCR; GFP fusion
A74ΔF4	GTATTGATTCTAATTTAGTTATG	PCR; GFP fusion
pGFPrev	TTATTTGTATAGTTCATCCATCCATGCC	PCR; GFP fusion
5′Biot-A74 pfullF	GGCAATGTTTGCTAAGGTG	EMSA

### Generation of transcriptional reporters and strains.

**FIG 9:**
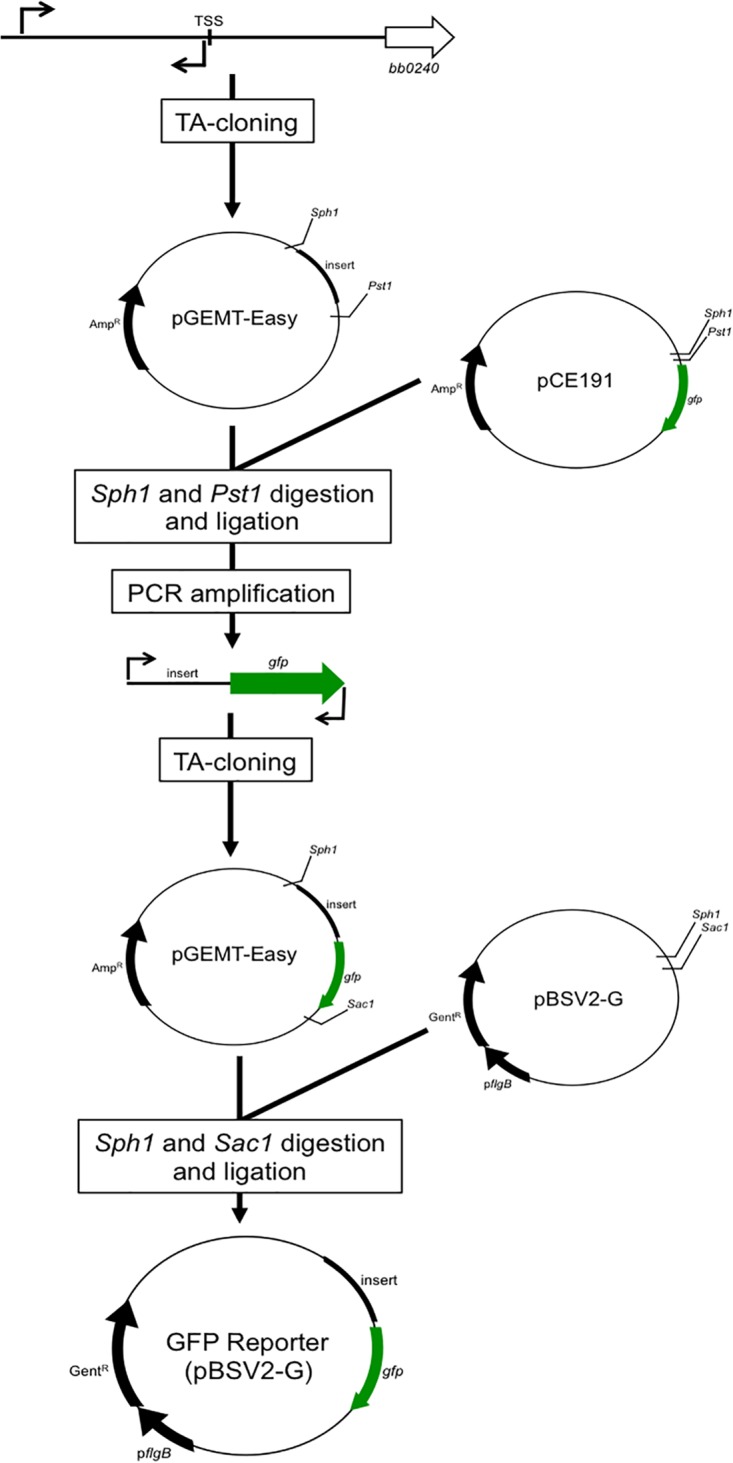
Scheme for generation of promoter-*gfp* transcriptional fusion reporter constructs. Cloning of the *glp* operon promoter is used for illustration.

Figure 9 contains a schematic describing the generation of the *gfp* transcriptional fusions for the *glp* operon, *ospAB*, and *bba74* upstream regions. Chromosomal DNA containing the region upstream of the relevant TSS was amplified from strain B31 5A18 NP1 (59) by PCR using primers listed in Table 1, cloned into pGEM-T Easy (Promega) according to the manufacturer’s instructions, and transformed into *E. coli* DH5α followed by blue/white selection on LB-ampicillin plates. Clones were confirmed by sequencing (Genewiz, South Plainfield, NJ) using pGEM-T universal forward and reverse primers. Insert orientation was determined by PCR and/or restriction enzyme digest. For the transcriptional fusions, the promoter regions of interest were amplified from each clone using pGEM-T Easy universal forward and reverse primers. Purified amplicons were digested with SphI (Fermentas, Pittsburgh, PA) and PstI (Fermentas) and ligated upstream of the promoterless *gfp* cassette in pCE191 (21) using the Rapid Ligation kit according to the manufacturer’s protocol (Denville, South Plainfield, NJ). Ligations were transformed into *E. coli* DH5α and selected on LB agar plates containing 100 μg/ml ampicillin. Clones were confirmed by PCR amplification and DNA sequencing. Promoter-*gfp* fusion cassettes were amplified from pCE191 using promoter-specific forward and pGFPrev primers (Table 1), cloned into pGEM-T Easy as described above, and subcloned into the *B. burgdorferi-E. coli* shuttle vector pBSV2-G (60) using SphI and SacI. Transformants were selected on LB agar plates containing 8 to 12 μg/ml of gentamicin. Clones were confirmed by PCR using the corresponding forward promoter primer and pGFPrev (Table 1).

Each transcriptional reporter construct in pBSV2-G was isolated and purified in a large-scale plasmid extraction by the alkaline lysis method (61). Thirty to 40 μg of plasmid DNA was electroporated into competent B31 5A18 NP1 (62), following which transformants were cultivated in 96-well plates in BSK-II containing 40 μg/ml of gentamicin and 100 μg/ml of kanamycin. For some experiments, reporter constructs were transformed into a strain 297 Δ*rpoS* mutant, CE174 (25), and cultivated in BSK-II containing 40 μg/ml of gentamicin and 0.06 μg/ml of erythromycin. Desired transformants were serially diluted and screened by PCR using the corresponding forward promoter primer and pGFPrev (Table 1).

### Cultivation of spirochetes in dialysis membrane chambers.

*B. burgdorferi* strains containing the transcriptional reporters were cultivated in DMCs (22, 36, 37). Cells were initially grown in BSK-II at 25°C to a density of 1 × 10^7^ cells/ml, following which they were transferred to fresh BSK-II at an initial density of 3,000 cells/ml and cultivated at 37°C to late logarithmic phase (~1 × 10^8^ cells/ml). Temperature-shifted organisms were used to inoculate 10 ml of BSK-II within a DMC (8,000-Da cutoff) at 3,000 cells/ml. Chambers were implanted in the peritoneal cavities of 160- to 200-g female Sprague-Dawley rats (Harlan, Chicago, IL). Two weeks postimplantation, DMCs were removed and their contents were transferred to a sterile 15-ml tube. Spirochete density was immediately determined by dark-field microscopy (63).

Mammalian host adaptation of DMC-cultivated spirochetes was assessed by real-time reverse transcription-quantitative PCR (qRT-PCR) measurement of transcripts for *ospC* and the endogenous copies of *ospA*, *glpF*, and *bba74* as described previously (28). Subsequent analyses used only samples that exhibited induction of *ospC* and repression of *ospA*, *glpF*, and *bba74*.

### Flow cytometry.

*B. burgdorferi* cells containing GFP transcriptional reporters were temperature shifted from 23°C to 37°C in BSK-II as described above and grown to a density of 1 × 10^7^ to 10 × 10^7^ cells/ml. A 1.0- to 1.5-ml amount of the culture was transferred to a microcentrifuge tube and centrifuged at 8,000 × *g* for 10 min. For DMC-cultivated *B. burgdorferi*, 5 to 10 ml of DMC contents (~5 × 10^7^ organisms) was centrifuged at 8,000 × *g* for 10 min. Cell pellets were resuspended in 500 μl of 2.5 μM SYTO 59 (Invitrogen) in TN buffer (10 mM Tris-HCl [pH 8.0], 1 mM EDTA, 100 mM NaCl) and incubated in the dark for 20 to 30 min at room temperature. The stained samples were washed twice in TN buffer, resuspended in 300 μl of 1% paraformaldehyde in 1× PBS, and stored in the dark at 4°C prior to analysis. Samples were analyzed by multiparameter flow cytometry using a MACSQuant Analyzer and MACSQuantify software (Miltenyi Biotec). Compensation for spectral overlap in the fluorescein isothiocyanate (FITC) (GFP) and allophycocyanin (APC) (SYTO 59) channels was performed using a GFP-expressing (no SYTO 59) strain and a non-GFP-expressing SYTO 59-stained strain (64). Fifty thousand events per sample were collected. Threshold values for GFP- and SYTO 59-positive cells were determined using unstained, nonfluorescent *B. burgdorferi* and SYTO 59-stained and unstained, GFP-expressing spirochetes. *B. burgdorferi* was gated to include only cells that stained with SYTO 59. The GFP (FITC) MFI of GFP-expressing SYTO 59^+^ cells was determined. For each experimental parameter, technical duplicates were prepared from two or three independent cultures, and the values for technical replicates were averaged. GFP MFIs of experimental groups were compared by a two-tailed, unpaired *t* test. Significance was defined as a *P* value of <0.05.

### EMSA.

Cell lysates (from 5 × 10^7^ to 7 × 10^7^ cells total) were obtained from either *B. burgdorferi* B31 5A18 NP1 or CE174 cultivated at 37°C or in DMCs as described above. Cells were pelleted by centrifugation, washed twice in PBS, and suspended in 0.3 ml of BugBuster protein extraction reagent (Novagen, Madison, WI), 10 µl phenylmethylsulfonyl fluoride, and 0.4 mg/ml lysozyme. Following incubation for 40 min on ice, extracts were cleared by centrifugation at 22,000 × *g* for 30 min, and protein concentration was determined using the Pierce Coomassie blue assay (Pierce Biotechnology, Rockford, IL). Cell lysates were stored at −80°C in aliquots of 0.5 μg/μl and used only once. A 283-bp biotin-labeled DNA target representing the region upstream of the *bba74* promoter was generated by PCR using primers 5′Biot-A74pfullF and A74pfullR (Table 1). Unlabeled competitor DNA was prepared by PCR using the same primer sequences, except that the forward primer was not biotinylated. Each EMSA was performed in a total volume of 20 μl containing 50 ng of poly(dI-dC), 50 fmol biotin-labeled target DNA, and 3 μg of protein lysate. In target competition reactions, an additional 4 pmol of unlabeled target DNA was added per reaction mixture. Protocols for the order of addition of reagents and reaction conditions followed those recommended by the manufacturer of the LightShift chemiluminescent EMSA kit (Thermo Fisher Scientific, Suwanee, GA). Since the order of addition of cell extracts and biotin-labeled target DNA may affect the specificity of the DNA-protein complexes, in all reaction mixtures that did not contain unlabeled competitor target DNA, components were premixed prior to adding the biotin-labeled target DNA. In reaction mixtures that did include competitive target DNA, unlabeled target DNA was added to the premixed components and chilled on ice for 20 min prior to the addition of biotin-labeled target DNA. All reaction mixtures were incubated at room temperature for 20 min. EMSA reactions were resolved by electrophoresis in 8% native polyacrylamide gels in 0.5× TBE (10 mM Tris-borate, 10 mM boric acid, 50 mM EDTA, pH 8.0) buffer at 200 V. Gels were blotted to charged nylon membranes (Hybond-N^+^; GE Healthcare, Buckinghamshire, United Kingdom) and cross-linked by UV light. Visualization of DNA bands was accomplished with the LightShift EMSA chemiluminescent kit.
